# Efficacy and Safety of Ibrutinib in Central Nervous System Lymphoma: A PRISMA-Compliant Single-Arm Meta-Analysis

**DOI:** 10.3389/fonc.2021.707285

**Published:** 2021-07-01

**Authors:** Liwei Lv, Xuefei Sun, Yuchen Wu, Qu Cui, Yuedan Chen, Yuanbo Liu

**Affiliations:** Department of Hematology, Beijing Tiantan Hospital, Capital Medical University, Beijing, China

**Keywords:** central nervous system lymphoma, ibrutinib, meta-analysis, single-arm, refractory, relapsed

## Abstract

**Background:**

Central nervous system lymphoma (CNSL) is an aggressive lymphoma. Studies investigating primary CNSL determined that the Bruton tyrosine kinase (BTK) played an important role in pathogenesis. Ibrutinib, an oral BTK inhibitor, is a new treatment strategy for CNSL. The purpose of this meta-analysis was to clarify the effectiveness and safety of ibrutinib in the treatment of CNSL.

**Methods:**

A systematic search of PubMed, Embase, Cochrane library, Wanfang Data Knowledge Service Platform, and China National Knowledge Infrastructure databases was conducted through to 31 October 2019. Studies involving patients with CNSL who received ibrutinib that reported the overall response (OR), complete remission (CR), and partial response (PR) were included. The random-effects or fixed-effects model with double arcsine transformation was used for the pooled rates and 95% confidence intervals (CI) were determined for all outcomes.

**Results:**

Eight studies including 162 patients were identified and included in the meta-analysis. The pooled OR rate after treatment with ibrutinib was 69% (95% CI, 61–79%, *I^2^* = 47.57%, *p* = 0.06), while the pooled CR and PR was 52% (95% CI, 35–68%, *I^2^* = 74.95%, *p* = 0.00) and 17% (95% CI, 7–30%, *I^2^* = 67.85%, *p* = 0.00), respectively. Among PCNSL patients, including new diagnoses PCNSL and R/R PCNSL, the pooled OR rate was 72% (95% CI, 63–80%, *I^2^* = 49.20%, *p* = 0.06) while the pooled CR and PR rates were 53% (95% CI, 33–73%, *I^2^* = 75.04%, *p* = 0.00) and 22% (95% CI, 14–30%, *I^2^* = 46.30%, *p* = 0.07), respectively. Common adverse events above grade 3 included cytopenia and infections.

**Conclusions:**

The ibrutinib-containing therapy was well tolerated and offered incremental benefit to patients with CNSL. However, randomized-controlled studies that directly compare efficacy and adverse events of ibrutinib are still needed.

**Systematic Review Registration:**

https://www.crd.york.ac.uk/prospero/, identifier CRD42020218974.

## Introduction

Central nervous system lymphoma (CNSL) includes primary and secondary lymphoma. Primary CNSL (PCNSL) is a rare form of aggressive extranodal non-Hodgkin lymphoma, which mainly affects the brain, spinal cord, meninges, and eyes. PCNSL is characterized by highly proliferative tumor cells, with a vascular-centric growth pattern, and diffuse infiltrate adjacent to central nervous system tissues (1). Approximately 90% of PCNSL pathology is represented by diffuse large B cell lymphoma (DLBCL), including three molecular subgroups, germinal center B-cell-like (GCB), activated B-cell-like (ABC) and type 3 subgroups (2). Secondary CNSL (SCNSL) refers to secondary involvement of the central nervous system, at presentation or at relapse in patients with systemic lymphoma (3). Regardless of whether the patient is diagnosed with PCNSL or SCNSL, treatment is very difficult, the patient’s prognosis is poor, and relapse is likely. Therefore, it is important to prolong progression-free survival (PFS) and treat refractory and relapsed (r/r) CNSL.

In recent years, studies on PCNSL have found that Bruton tyrosine kinase (BTK) plays an important role in regulating the oncogenic signal transduction downstream of B-cell antigen receptor (BCR) and Toll-like receptor (TLR) (4). Ibrutinib, an oral irreversible inhibitor of BTK, is considered to be effective for the treatment of CNSL, especially r/r CNSL (5); however, the exact efficacy and safety of ibrutinib in the treatment of CNSL is still unclear. The purpose of this meta-analysis was to study the effectiveness and safety of ibrutinib-based treatment in CNSL patients. The results of this study may have a guiding tole for clinical treatment.

## Methods

### Data Sources and Literature Searches

This study was conducted following the Preferred Reporting Items for Systematic Reviews and Meta-Analyses (PRISMA) guidelines (6, 7). Three investigators independently searched for studies published before 30 October 2020 in PubMed, Embase, Cochrane library, Wanfang Data Knowledge Service Platform, and the China National Knowledge Infrastructure databases. The search keywords were “central nervous system lymphoma” and “ibrutinib”; the search strategy for each database is shown in **Supplementary Table 1**. The search was not restricted by region, race, age, or payment method. In addition, references to reviews and original studies were scanned to avoid missing any studies that should be included. The databases were searched and results imported to EndNote software (X8 version). The software consolidates the results of the database and identifies duplicate articles.

### Selection Criteria

The inclusion criteria were as follows: (1) prospective studies and retrospective studies (including randomized control trials, cohort studies, single-arm studies); (2) studies including patients confirmed with CNSL, irrespective of the primary or secondary nature; (3) studies including patients treated with ibrutinib, both as monotherapy and in combination with other agents; (4) studies reporting efficacy end points, including the overall response (OR), complete response (CR), and partial response (PR).

The exclusion criteria were as follows: (1) incomplete data for the targeted outcomes; (2) reported outcomes from multiple populations or disease cohorts; (3) conference abstracts, reviews, comments, case reports, cases that reported incomplete information, and cellular or animal studies.

Two researchers independently reviewed the title and abstract of the study and submit eligible studies to full-text analysis to confirm whether they should be included in the meta-analysis. After each selection stage, the two researchers compared their findings. Any inconsistency was resolved and discussed with a third researcher.

### Quality Assessment

Prospective non-randomized studies (single-arm studies) were assessed by methodological index for non-randomized studies (MINORS) (8). The retrospective studies without a comparison group were assessed by JBI Critical Appraisal Checklist for Case Series (9).

### Data Extraction

Two researchers independently conduct data extraction, and any differences in opinion were resolved in participation with the third author in a joint discussion.

The following data were extracted from each study: the first author’s name, year of publication, study design, median follow-up time, disease status, sample size, median age, sex of patients, treatment, and main outcomes. The main outcomes included OR, CR, and PR according to the International Primary CNS Lymphoma Collaborative Group criteria (10). The following data were also extracted if the study contains: PFS, overall survival (OS), and adverse events (AEs). PFS was defined as time from initiation of ibrutinib to disease progression, death from any cause, or last follow up. OS was defined as time from initiation of ibrutinib to death from any cause or last follow-up. AEs were classified by the Common Terminology Criteria for Adverse Events version 4. While original survival data were hardly accessed, the extracted data from the Kaplan-Meier curves (K-M curves) were obtained by software Engauge Digitizer version 11.1.

### Statistical Analysis

All data analyses were performed using the STATA SE14.0 (StataCorp, College Station, TX, USA). The pooled rates used a random effects model or a fixed effect model with double arcsine transformation. The effect size of all combined results is represented by the 95% CI (with upper and lower limits). Cochran’s Q test and *I^2^* statistics were used to assess the heterogeneity between studies. The fixed-effects model was used for pooled results with low heterogeneity (*I^2^* ≤50%); otherwise, the random-effects model was used for analysis. Sensitivity analysis was performed by excluding each study one by one from the pooled results with high heterogeneity. Moreover, potential publication bias of included studies was examined using the Begg’s and Egger’s tests.

## Results

### Study Selection and Characteristics

**Figure 1:**
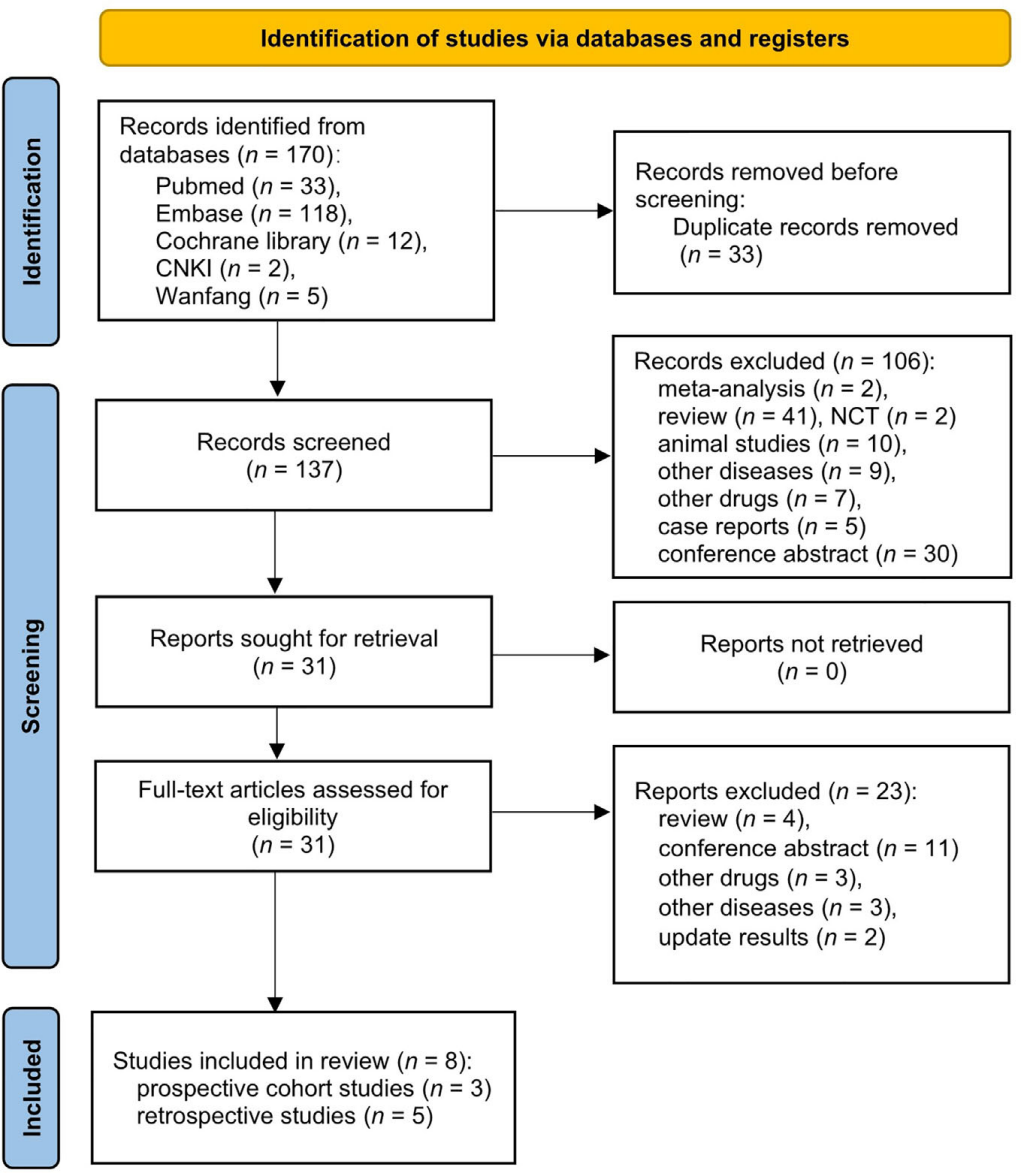
Flow diagram of study selection.

In the initial search, 170 relevant articles were identified (163 English articles and 7 Chinese articles). After the exclusion of 33 duplicate articles using EndNote X8 software, 137 articles underwent a title and abstract review. A total of 106 studies were excluded for following reasons: animal studies (*n* = 10), other drugs (*n* = 7), other diseases (*n* = 9), conference abstracts (*n* = 30), case reports (*n* = 5), reviews (*n* = 41), meta-analyses (*n* = 2), and National Clinical Trial registration (*n* = 2). For 31 articles, the full text was reviewed and 23 of them were excluded for following reasons: conference abstract (*n* = 11), other drugs (*n* = 3), other diseases (*n* = 3), review (*n* = 4), update of results (*n* = 2). The eight remaining studies fulfilled the eligibility criteria and were included in the meta/analysis and included three prospective cohort studies (11–13) and four retrospective studies (14–18). The literature review and identification process are shown in **Figure 1**. The meta/analysis evaluated the efficacy and toxicity of the ibrutinib regimen for a total of 162 patients with CNSL across eight cohort studies (11–18). The age of the patients ranged from 18 to 87 years, with a male predominance (58.64%). Baseline clinical characteristics of these patients are summarized in **Table 1**.

**Table 1:** Baseline clinical characteristics of included studies.

Study	Country	Design	Study period	Median follow-up time, months (range)	Disease status	Sample size	Median age, years (range)	Sex male/female	Intervention	End points
14	Australia	Retrospective	Before 2/2019	16.6 (0.5–61.5)	PCNSL/SCNSL (previously untreated/r/r)	33	64 (22–85)	23/10	Ibrutinib ± radiotherapy/chemotherapy	OR, CR, PR, OS, PFS, AEs
15	Germany	Retrospective	12/2017–1/2020	14.2 (2.5–23.7)	r/r CNSL	9	63 (53–82)	7/2	Ibrutinib ± radiotherapy/chemotherapy	OR, CR, PR, OS, PFS
16	China	Retrospective	12/2018–6/2019	11.6	PCNSL (previously untreated)	11	56 (41–68)	7/4	Ibrutinib+ HD-MTX	OR, CR, PR, OS, PFS, AEs
11	France	Prospective, open-label, multicenter, phase II	9/2015–7/2016	25.7	r/r PCNSL	44	70 (52–81)	20/24	Ibrutinib	OR, CR, PR, OS, PFS, AEs
12	America	Prospective, open-label, single-center, phase Ib	NR	19.7 (12.7–27.1)	r/r CNSL	15	62 (23–74)	8/7	Ibrutinib+ HD-MTX ± R	OR, CR, PR, OS, PFS, AEs
13	America	Prospective, phase Ib	8/2014–3/2016	15.5 (8–27)	PCNSL (previously untreated/r/r)	18	66 (49–87)	11/7	DA-TEDDi-R	OR, CR, PR, AEs
17	France and Belgium	Retrospective	4/2015–4/2016	NR	r/r CNSL	14	68 (48–79)	9/5	Ibrutinib	OR, CR, PR
18	China	Retrospective	9/2017–12/2019	13 (3–27)	r/r PCNSL	18	58.5 (18–76)	10/8	Ibrutinib+ MIDD	OR, CR, PR, OS, PFS, AEs

r/r, relapsed/refractory; PCNSL, primary central nervous system lymphoma; SCNSL, secondary central nervous system lymphoma; HD-MTX, high-dose methotrexate; R, rituximab; DA-TEDDi-R, ibrutinib, pegfilgrastim, cytarabine, temozolomide, etoposide, liposomal doxorubicin, dexamethasone, and rituximab; MIDD, high-dose methotrexate, ifosfamide, liposomal doxorubicin, methylprednisolone; OR, overall response; CR, complete response; PR, partial response; OS, overall survival; PFS, progression-free survival; AEs, adverse event; NR, not reported.

### Quality Assessment

Three single-arm studies assessed using the MINORS index score ranged from 12 to 15 points, which was acceptable for the present meta-analysis (**Table 2A**). Five retrospective studies without comparison were included after they were assessed using the JBI Critical Appraisal Checklist for Case Series (**Table 2B**).

**Table 2:** Quality assessment of included studies.

A. MINORS index for included non-randomized studies									
Study	I	II	III	IV	V	VI	VII	VIII	Total
Soussain 2019	2	2	2	2	1	2	2	2	15
Grommes 2019	2	2	2	2	0	2	2	2	14
Lionakis 2017	2	1	2	2	2	1	2	0	12

Numbers I–Ⅷ in heading signified: Ⅰ, a clearly stated aim; Ⅱ, inclusion of consecutive patients; Ⅲ, prospective collection of data; Ⅳ, endpoints appropriate to the aim of the study; Ⅴ, unbiased assessment of the study endpoint; Ⅵ, follow-up period appropriate to the aim of the study; Ⅶ, loss of follow up less than 5%;Ⅷ, prospective calculation of the study size.

### Efficacy

#### Tumor Response

**Figure 2:**
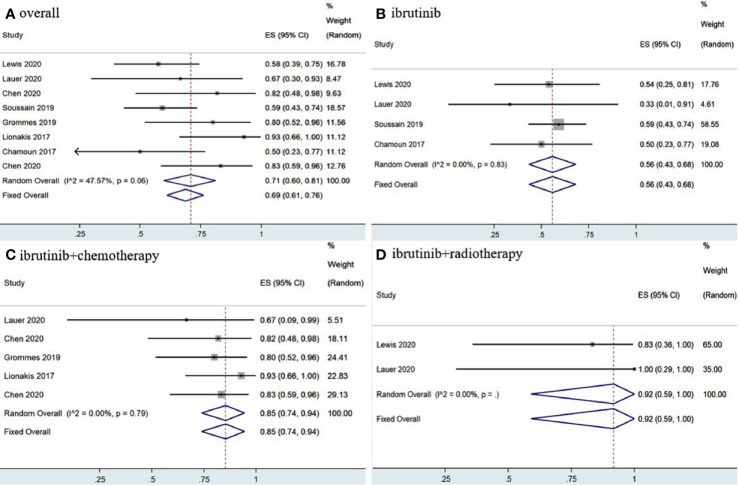
Pooled overall response of central nervous system lymphoma. **(A)** Ibrutinib-based regimens. **(B)** Ibrutinib monotherapy. **(C)** Ibrutinib combined with chemotherapy. **(D)** Ibrutinib combined with radiotherapy.

**Figure 3:**
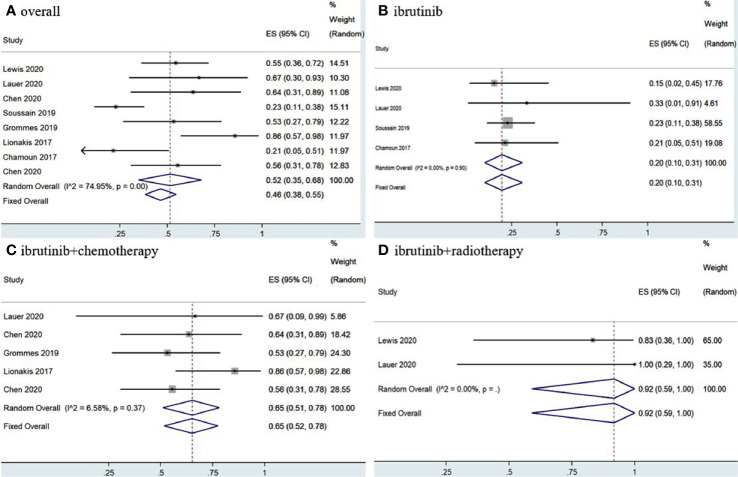
Pooled complete response rates of central nervous system lymphoma. **(A)** Ibrutinib-based regimens. **(B)** Ibrutinib monotherapy. **(C)** Ibrutinib combined with chemotherapy. **(D)** Ibrutinib combined with radiotherapy.

The eight included studies reported OR, CR, and PR as clinical outcomes. The pooled OR rate after treatment with ibrutinib was 69% (95% CI, 61–76%, *I^2^* = 47.57%, *p* = 0.06) while the pooled CR and PR rates were 52% (95% CI, 35–68%, *I^2^* = 74.95%, *p* = 0.00) and 17% (95% CI, 7–30%, *I^2^* = 67.85%, *p* = 0.00), respectively (**Figures 2**, **3** and **Supplementary Figure 2**).

Group analysis of ibrutinib combined with different treatment measures showed that the pooled OR, CR, and PR rates of different treatment strategies were different. Ibrutinib monotherapy was given in four studies (11, 14, 15, 17), and the pooled OR rate was 56% (95% CI, 43–68%, *I^2^* = 0.00%, *p* = 0.83), while the pooled CR and PR rates were 20% (95% CI, 10–31%, *I^2^* = 0.00%, *p* = 0.90) and 32% (95% CI, 21–44%, *I^2^* = 0.00%, *p* = 0.53), respectively (**Figures 2**, **3** and **Supplementary Figure 3**). Moreover, some chemotherapy regimens (12, 13, 15, 16, 18), containing rituximab, HD-MTX (high-dose methotrexate) ± rituximab, Dara, DA-TEDDi-R (pegfilgrastim, cytarabine, temozolomide, etoposide, liposomal doxorubicin, dexamethasone, and rituximab), and MIDD (high-dose methotrexate, ifosfamide, liposomal doxorubicin, methylprednisolone) combined with ibrutinib in patients with CNSL resulted in a pooled OR of 85% (95% CI, 74–94%, *I^2^* = 0.00%, *p* = 0.79), while the pooled CR and PR rates were 65% (95% CI, 52–78%, *I^2^ =* 6.58%, *p* = 0.37) and 18% (95% CI, 8–29%, *I^2^* = 0.00%, *p* = 0.53), respectively (**Figures 2**, **3** and **Supplementary Figure 3**). Only two studies (14, 15) treated patients with ibrutinib combined with radiotherapy, and the pooled OR and CR was 92% (95% CI, 59–100%, *I^2^* = 0.00%) (**Figures 2**, **3**).

**Figure 4:**
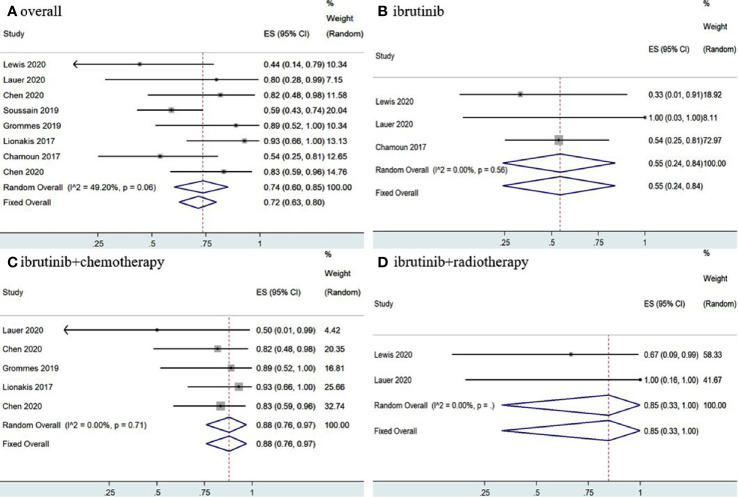
Pooled complete response rates of primary central nervous system lymphoma. **(A)** Ibrutinib-based regimens. **(B)** Ibrutinib monotherapy. **(C)** Ibrutinib combined with chemotherapy. **(D)** Ibrutinib combined with radiotherapy.

**Figure 5:**
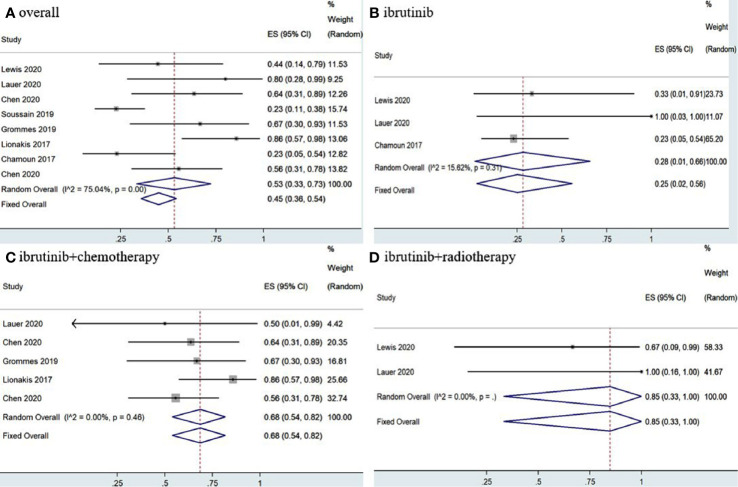
Pooled complete response rates of primary central nervous system lymphoma. **(A)** Ibrutinib-based regimens. **(B)** Ibrutinib monotherapy. **(C)** Ibrutinib combined with chemotherapy. **(D)** Ibrutinib combined with radiotherapy.

Statistical analysis of PCNSL patients showed that the pooled OR rate was 72% (95% CI, 63–80%, *I^2^* = 49.20%, *p* = 0.06) while the pooled CR and PR rates were 53% (95% CI, 33–73%, *I^2^* = 75.04%, *p* = 0.00) and 22% (95% CI, 14–30%, *I^2^* = 46.30%, *p* = 0.07), respectively (**Figures 4**, **5** and **Supplementary Figure 4**). Ibrutinib monotherapy was used in three studies (14, 15, 17), and the pooled OR was 55% (95% CI, 24–84%, *I^2^* = 0.00%, *p* = 0.56), while the pooled CR and PR rates were 25% (95% CI, 2–56%, *I^2^* = 15.62%, *p* = 0.31) and 16% (95% CI, 0–45%, *I^2^* = 0.00%, *p* = 0.50), respectively (**Figures 4**, **5** and **Supplementary Figure 5**). Five studies (12, 13, 15, 16, 18) used ibrutinib combined with chemotherapy to PCNSL patients, and the pooled OR was 88% (95% CI, 76–97%, *I^2^* = 0.00%, *p* = 0.71), while the pooled CR and PR rates were 68% (95% CI, 54–82%, *I^2^* = 0.00%, *p* = 0.46) and 16% (95% CI, 5–29%, *I^2^* = 0.00%, *p* = 0.66), respectively (**Figures 4**, **5** and **Supplementary Figure 5**). Only two studies (14, 15) evaluated ibrutinib combined with radiotherapy, and the pooled OR and CR was 85% (95% CI, 33–100%, *I^2^* = 0.00%) (**Figures 4**, **5**).

**Figure 6:**
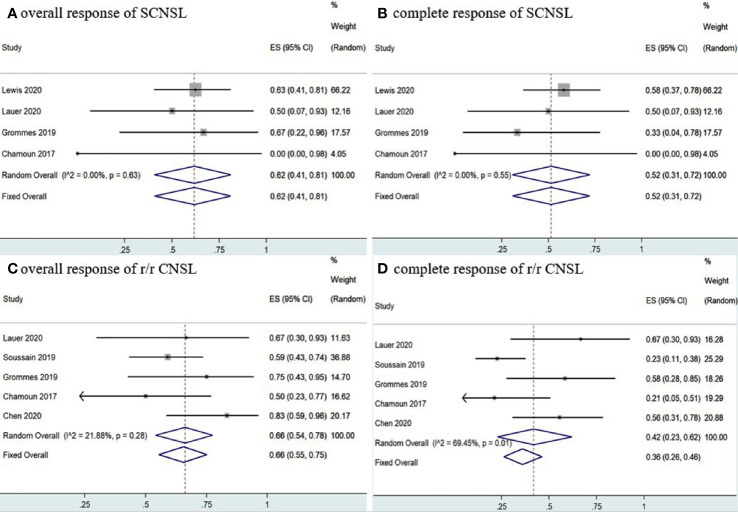
Pooled response rates of secondary central nervous system lymphoma (SCNSL) and refractory/relapsed central nervous system lymphoma (r/r CNSL). **(A)** Pooled overall response of SCNSL. **(B)** Pooled complete response of SCNSL. **(C)** Pooled overall response of r/r CNSL. **(D)** Pooled complete response of r/r CNSL.

A total of 35 SCNSL patients were enrolled in four studies (12, 14, 15, 17), the pooled OR rate after treatment with ibrutinib-based treatment was 62% (95% CI, 41–81%, *I^2^* = 0.00%, *p* = 0.63) while the pooled CR and PR rates were 52% (95% CI, 31–72%, *I^2^* = 0.00%, *p* = 0.55) and 1% (95% CI, 0–13%, *I^2^* = 11.22%, *p* = 0.34), respectively (**Figure 6** and **Supplementary Figure 6**).

**Figure 7:**
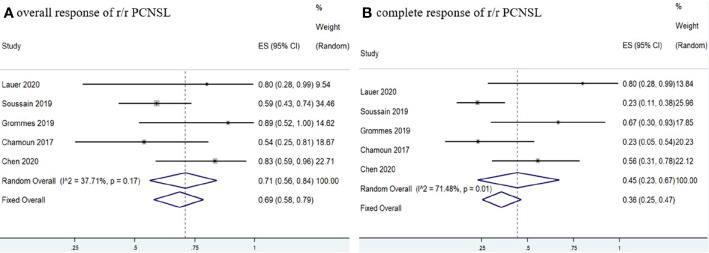
Pooled response rates of refractory/relapsed primary central nervous system lymphoma (r/r PCNSL). **(A)** Pooled overall response of r/r PCNSL. **(B)** Pooled complete response of r/r PCNSL.

Five studies (11, 12, 15, 17, 18) investigated 97 r/r CNSL patients treated with ibrutinib, and the pooled OR was 66% (95% CI, 55–75%, *I^2^* = 21.88%, *p* = 0.28), while the pooled CR and PR rates were 42% (95% CI, 23–62%, *I^2^* = 69.45%, *p* = 0.01) and 23% (95% CI, 10–37%, *I^2^* = 50.59%, *p* = 0.09), respectively (**Figure 6** and **Supplementary Figure 6**). A total of 89 PCNSL patients were included in r/r CNSL, the pooled OR rate after treatment with ibrutinib was 69% (95% CI, 58–79%, I2 = 37.71%, P = 0.17) while the pooled CR and PR was 45% (95% CI, 23–67%, I2 = 71.48%, P = 0.01) and 29% (95% CI, 19–40%, I2 = 0.99%, P = 0.40), respectively (**Figure 7** and **Supplementary Figure 7**).

**Figure 8:**
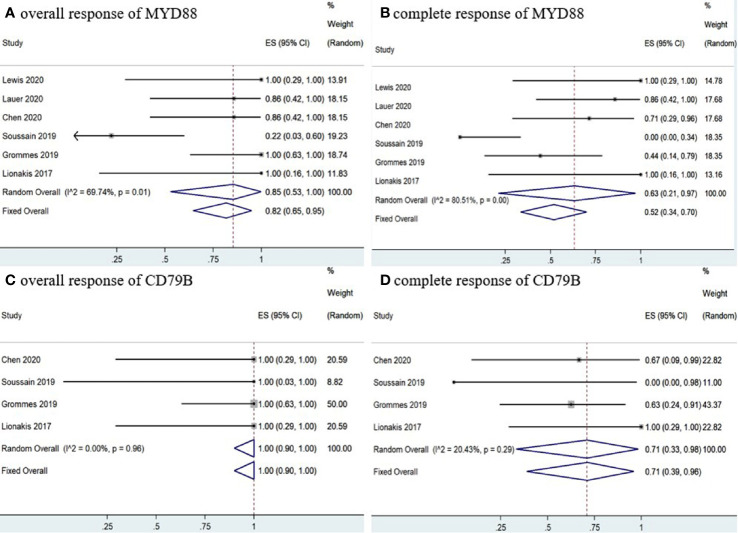
Pooled response rates of *MYD88* mutation and *CD79B* mutation. **(A)** Pooled overall response rate of *MYD88* mutations. **(B)** Pooled complete response rate of *MYD88* mutations. **(C)** Pooled overall response of *CD79B* mutations. **(D)** Pooled complete response of *CD79B* mutations.

**Figure 9:**
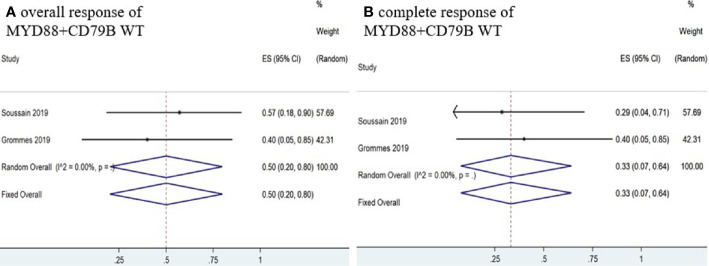
Pooled response rate for *MYD88* and *CD79B* wild-type gene status. **(A)** Pooled overall response of *MYD88* and *CD79B* wild-type. **(B)** Pooled complete response of *MYD88* and *CD79B* wild-type.

Six studies (11–16) determined *MYD88* mutation status in patients and the pooled OR of patients with *MYD88* mutation who received ibrutinib treatment was 85% (95% CI, 53–100%, *I^2^* = 69.74%, *p* = 0.01), while the pooled CR and PR rates were 63% (95% CI, 21–97%, *I^2^* = 80.51%, *p* = 0.00) and 16% (95% CI, 4–32%, *I^2^* = 41.64%, *p* = 0.13), respectively (**Figure 8** and **Supplementary Figure 8**). In addition, four studies (11–13, 16) monitored *CD79B* mutational status. The pooled OR of patients with *CD79B* mutation was 100% (95% CI, 90–100%, *I^2^* = 0.00%, *p* = 0.96), while the pooled CR and PR rates were 71% (95% CI, 39–96%, *I^2^* = 20.43%, *p* = 0.29) and 29% (95% CI, 4–61%, *I^2^* = 20.43%, *p* = 0.29), respectively (**Figure 8** and **Supplementary Figure 8**). The pooled OR rate of patients with both *MYD88* and *CD79B* wild-type status who received ibrutinib treatment was 50% (95% CI, 20–80%, *I^2^* = 0.00%), while the pooled CR and PR rates were 33% (95% CI, 7–64%, *I^2^* = 0.00%) and 13% (95% CI, 0–40%, *I^2^* = 0.00%), respectively (**Figure 9** and **Supplementary Figure 7**).

#### Survival

**Figure 10:**
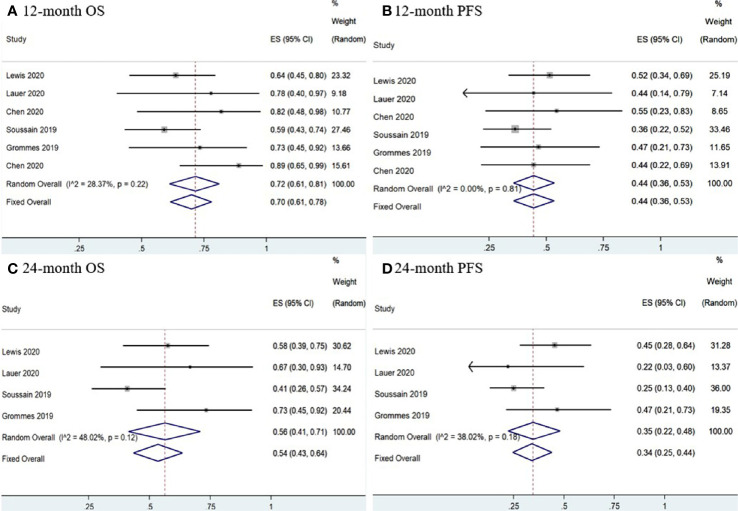
Survival of central nervous system lymphoma. **(A)** 12-mouth overall survival. **(B)** 12-mouth progression-free survival. **(C)** 24-mouth overall survival. **(D)** 24-month progression-free survival.

Six studies (11, 12, 14–16, 18) had complete PFS and OS K-M curves. The 12-and 24-month pooled PFS rates for CNSL patients treated with ibrutinib were 44% (95% CI, 36–53%, *I^2^* = 0.00%, *p* = 0.81) and 34% (95% CI, 25–44%, *I^2^* = 38.02%, *p* = 0.18), respectively (**Figure 10**). The 12- and 24-month pooled OS rates were 70% (95% CI, 61–78%, *I^2^* = 28.37%, *p* = 0.22) and 54% (95% CI, 43–64%, *I^2^* = 48.02%, *p* = 0.12), respectively (**Figure 10**).

Three studies (11, 15, 18) reported PFS and OS K-M curves in r/r CNSL patients. The 12-and 24-month pooled PFS rates for r/r CNSL patients treated with ibrutinib were 39% (95% CI, 28–51%, *I^2^* = 0.00%, *p* = 0.79) and 24% (95% CI, 13–37%, *I^2^* = 0.00%), respectively (**Supplementary Figure 9**). The 12- and 24-month pooled OS rates were 75% (95% CI, 52–92%, *I^2^* = 65.37%, *p* = 0.06) and 45% (95% CI, 31–59%, *I^2^* = 0.00%), respectively (**Supplementary Figure 9**).

### Toxicity

AEs were reported in six studies (11–14, 16, 18). Hematological AEs mainly included neutropenia, anemia, and thrombocytopenia. The pooled rate of grade 3–4 neutropenia toxicity was 8% (95% CI, 3–15%, *I^2^* = 0.89%, *p* = 0.36), grade 3–4 anemia toxicity was 7% (95% CI, 1–16%, *I ^2^ =* 0.00%), grade 3–4 thrombocytopenia toxicity was 6% (95% CI, 1–15%, *I^2^* = 0.00%, *p* = 0.88). Severe non-hematological AEs mainly included infection, febrile neutropenia, bleeding, atrial fibrillation, and liver damage. The pooled rate of grade 3–4 infection was 11% (95% CI, 6–18%, *I^2^* = 34.30%, *p* = 0.19) while the pooled rate of grade 3–4 aspergillus infection was 3% (95% CI, 0–9%, *I^2^* = 21.65%, *p* = 0.28). The pooled rate of grade 3–4 febrile neutropenia was 4% (95% CI, 0–10%, *I^2^* = 0.00%). The pooled rate of grade 3–4 bleeding, atrial fibrillation, and alanine aminotransferase increased was 4% (95% CI, 0–10%, *I^2^* = 0.00%), 3% (95% CI, 0–8%, *I^2^* = 0.00%), and 5% (95% CI, 0–12%, *I^2^* = 0.00%), respectively (**Table 3**).

**Table 3:** Pooled results of common AEs of ≥grade 3.

Adverse Event	≥Grade 3	
	effect size, % (95% CI)	*I^2^*, %
Neutropenia	8 (3–15)	0.89
Anemia	7 (1–16)	0.00
Thrombocytopenia	6 (1–15)	0.00
Infection	11 (6–18)	34.30
Aspergillus infection	3 (0–9)	21.65
Febrile neutropenia	4 (0–10)	0.00
Bleeding	4 (0–10)	0.00
Atrial fibrillation	3 (0–8)	0.00
Alanine aminotransferase increased	5 (0–12)	0.00

### Sensitivity Analysis

**Figure 11:**
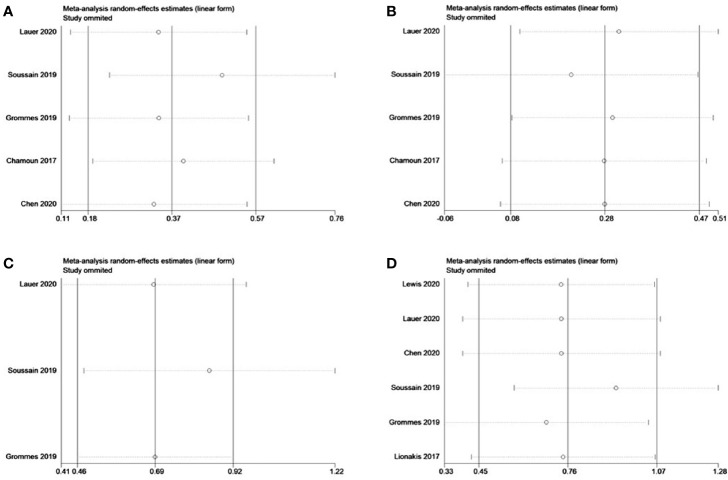
Sensitivity analysis of refractory/relapsed central nervous system lymphoma (r/r CNSL) and *MYD88* mutation. **(A)** Complete response of r/r CNSL. **(B)** Partial response of r/r CNSL. **(C)** 12-mouth overall survival of r/r CNSL. **(D)** Overall response of *MYD88* mutation.

Sensitivity analysis was performed by removing individual studies one by one from the pooled results with high heterogeneity. The pooled analysis of r/r CNSL and *MYD88* mutations did not change significantly when studies were omitted, indicating that our combined results are reliable (**Figure 11**).

### Publication Bias

Egger’s and Begg’s tests were performed to identify publication bias in this study. Assessment results of the pooled OR did not show significant publication bias among included studies, with P = 0.190 for the Egger’ test and *p* = 1.000 for Begg’s test. Similarly, Egger’s test (*p* = 0.945) and Begg’s test (*p* = 1.000) for AEs (grade 3–4) in total did not identify any publication bias with regard to safety outcome.

## Discussion

In recent years, with the continuous accumulation of PCNSL research, a variety of gene mutations and signaling pathways have been identified that are believed to play a role in the pathogenesis of PCNSL. *MYD88* is the most common mutation in PCNSL. The signal transduction protein encoded by *MYD88* stimulates TLR to induce activation of the NF-κB and JAK/STAT3 signaling pathways (19). *CD79B* is another common mutation. *CD79B* activates the NF-kB signaling pathway through the BCR signaling (19). The activation of both TLR and BCR pathways will lead to strong NF-κB activity (4). BTK is an important intermediate link between BCR/TLR and NF-κB (20). This provides a theoretical basis for the treatment of PCNSL with BTK inhibitors.

Ibrutinib is a small molecule inhibitor that can efficiently bind to the active site Cys-481 of BTK and reduces its activation (21). BTK is a member of the Tec kinase family and plays an important role in the BCR signaling pathway of B cells (22, 23). When inhibiting BTK, ibrutinib also inhibits or down-regulates BTK-related downstream signaling molecules (24). Several *in vivo* experiments have confirmed that ibrutinib participates in the regulation of the tumor microenvironment, and down-regulates the expression of chemokines and inflammatory cytokines (25), and inhibits the pathways that promote tumor cell activation and proliferation in the tumor microenvironment (26). More importantly, studies have confirmed that ibrutinib can quickly cross the blood-brain barrier (27). Therefore, ibrutinib as an oral BTK inhibitor has become a rational choice for the treatment of CNSL.

In this study, the OR rate of ibrutinib-based treatment for CNSL was 69%, and the rates of CR and PR were 52 and 17%, respectively. However, the *I^2^* values for overall CR and PR were 74.95 and 67.85%, which was quite heterogeneous. The subgroup analysis stratified according to the different treatment regimens including ibrutinib significantly reduced heterogeneity, indicating that the heterogeneity derived from the differences in the treatment plan. Subgroup analysis showed that the OR, CR, and PR rates from ibrutinib monotherapy for CNSL were 56, 20, and 32%, respectively. The OR, CR, and PR rates of ibrutinib combined with chemotherapy were 85, 65, and 18%, respectively. The OR and CR rates of ibrutinib combined with radiotherapy for CNSL both were 92%. The above results indicated that ibrutinib had a certain effect on the treatment of CNSL, and the efficacy of ibrutinib combined with chemotherapy or radiotherapy was superior to ibrutinib monotherapy, suggesting the addition of ibrutinib to the traditional treatment plan may improve efficacy.

At present, in the treatment of PCNSL, HD-MTX-based chemotherapy can achieve an OR of more than 60%, whereas CR can reach 40% or higher. In the IELSG20 randomized controlled trial, the OR and CR of HD-MTX combined with cytarabine for the treatment of PCNSL were 69 and 46%, respectively (28). In a study of elderly PCNSL patients with a median age of 71 years, the OR of the MTX-based treatment plan could reach more than 60% (29). In the IELSG32 randomized controlled study, the OR and CR of HD-MTX/cytarabine/rituximab for the treatment of PCNSL were 73 and 30%, and the HD-MTX/cytarabine/thiotepa combination for the treatment of PCNSL achieved a superior curative effect with OR and CR rates of 86 and 49% respectively (30). Our analysis of the efficacy of ibrutinib in the treatment of PCNSL patients showed that the pooled OR and CR rates could reach 72 and 53%, respectively. This showed that ibrutinib could achieve satisfactory results for the treatment of PCNSL. Thus, the efficacy of ibrutinib as a single agent compared with HD-MTX-based chemotherapy did not appear to be optimal. Nonetheless, the pooled OR and CR rates of ibrutinib combined with chemotherapy were as high as 88 and 68%, which appeared to have a better curative effect than the HD-MTX/cytarabine/rituximab combination. This suggests that for eligible patients, ibrutinib can be added to HD-MTX-based chemotherapy to achieve the purpose of improving the remission rate.

Although the combination of ibrutinib and radiotherapy also shows a very high remission rate, both OR and CR were 85%, in fact only two studies have reported the efficacy of ibrutinib combined with radiotherapy, and the number of cases is small, so its exact effect requires further studies by expanding the sample size. In addition, the meta-analysis by Hou et al. reported that the OR and CR rates for ibrutinib in the treatment of DLBCL were 57.9 and 35%, respectively (9). Among these, the OR and CR of ibrutinib monotherapy were 41.6 and 15.2%, respectively, and the OR and CR of ibrutinib combined with the rituximab-based chemotherapy were 72 and 47.5%, respectively (9). Our systematic analysis of PCNSL showed that the remission rate of ibrutinib for the treatment of PCNSL was higher than that of systemic DLBCL. Next-generation sequencing revealed that PCNSL patients had a different gene expression profile when compared to other types of DLBCL. Compared with ABC-DLBCL outside the brain, the frequency of *MYD88* and *CD79B* mutations in PCNSL is much higher (31). Therefore, the therapeutic targets of ibrutinib in PCNSL may be more abundant, leading to a better curative effect of ibrutinib in the treatment of PCNSL.

The team led by Professor Liu once reported a retrospective analysis of 19 cases of SCNSL treated with the R-MIADD (rituximab, high-dose methotrexate, ifosfamide, cytarabine, liposomal formulation of doxorubicin, and dexamethasone) regimen (32). The results showed that the OR and CR were 68.4 and 57.9%, respectively (32). A prospective multicenter phase II trial conducted by Korfel et al. included 30 patients with SCNSL and adopted the HD-MTX/ifosfamide/intrathecal liposomal cytarabine and HD-Ara-C/thiotepa/intrathecal liposomal cytarabine regimens to induce remission and patients who responded to treatment were subjected to high dose autologous stem cell transplantation. The final OR and CR were 71 and 63%, respectively (33). Our study showed that the pooled OR and CR rates of SCNSL patients treated with ibrutinib were 62 and 52%, respectively. Thus, although the remission rate of ibrutinib in the treatment of SCNSL was lower than that of multi-drug combination chemotherapy and ASCT, ibrutinib also achieved a good performance.

Our meta-analysis of studies in r/r CNSL showed the pooled OR, CR, and PR were 66, 42, and 23%, respectively. This indicated that ibrutinib was a treatment option for patients with r/r CNSL. However, due to the limited study results published to date, it is impossible to conduct a meta-analysis evaluating ibrutinib as a monotherapy or in combination. In the clinic, how to choose treatment options for patients with r/r CNSL is still being explored.

In this study, we also compared the efficacy of ibrutinib in patients with either a *MYD88* or *CD79B versus* wild-type gene status. Our findings showed that regardless of whether there was a *MYD88* or *CD79B* mutation, ibrutinib achieved considerable curative effect. We suspect that the possible reason is that ibrutinib is not only an irreversible BTK inhibitor, as increasing evidence *in vivo* and *in vitro* indicate, it is also an inhibitor of IL2-inducible T cell kinase (ITK), which can provide a wider range of alternative therapeutic effects as an immunomodulator (5). Thus, ibrutinib can also influence other immune cells and kinases, induce CD4+ T cells to differentiate into helper T cells (Th1), and enhance tumor immune surveillance (34). Therefore, for CNSL patients without *MYD88* or *CD79B* mutations, ibrutinib is also an alternative treatment strategy.

CNSL is characterized by poor prognosis and a high recurrence rate. Therefore, how to reduce the recurrence rate and prolong the survival of these patients has always been a difficult problem facing clinicians. Houillier et al. reported the survival time of HD-MTX-based systemic chemotherapy for PCNSL (35). The 1- and 2-year OS were 62 and 51%, respectively, and the 2-year PFS was 36% (35). Khimani et al. used radiotherapy as a rescue treatment for patients with r/r CNSL, and the 2-year OS was 32% (36). In this study, the 12- and 24-month OS of patients who received the ibrutinib-based treatment regimen were 70 and 54%, and the 12- and 24-month PFS were 44 and 34%, respectively. For r/r CNSL patients, 12- and 24-month OS and PFS were 75, 45, and 39, 24%, respectively. In terms of survival, ibrutinib can also benefit r/r CNSL patients, but it does not seem to have much advantage over HD-MTX-based chemotherapy. However, the follow-up times included in the literature analyzed in this study was relatively short, and most studies did not reach the median OS. Due to the propensity for recurrence of CNSL, the effects of ibrutinib on survival need to be further evaluated by extending the follow-up time.

We conducted a meta-analysis of grade 3–4 AEs and found that the most common AEs were hematological AEs and infections. hematological AEs are mainly manifested as cytopenia, including neutropenia, anemia, and thrombocytopenia, with an incidence of 6–8%. The highest incidence of non-hematological AEs was infection, which was 11%. Some studies have found that ibrutinib may cause severe Aspergillus infection and atrial fibrillation (37, 38). In this study, the pooled grade 3–4 Aspergillus infection and atrial fibrillation rate was only 4%. Nevertheless, the results of our study still suggested that clinicians should pay attention to the prevention of AEs such as bone marrow suppression, infection, arrhythmia, and liver damage when treating patients with ibrutinib.

The results of this study indicate that ibrutinib is safe and effective in the treatment of PCNSL. However, there are still many shortcomings: (1) The currently available clinical studies report single-arm studies with small sample sizes. The single-arm trials make difficult strong comparisons with other treatment options. According to the 1-year OS of patients, a two-sided, one-sample log-rank test calculated from a sample of 174 subjects achieves 80.1% power at a 0.050 significance level (39, 40). Therefore, we look forward to clinical trials with larger sample sizes in the future. (2) Most studies with published results are retrospective studies, and most prospective studies are phase I or II clinical trials. (3) The follow-up time is shorter. (4) Ibrutinib is currently used in r/r CNSL. Few data are available for ibrutinib as a first-line treatment for CNSL. The timing of medications that will bring the greatest benefit to patients is still unclear. (5) Due to language limitations, this study only included studies in English and Chinese.

## Conclusions

The pathogenesis of CNSL is unknown and there are obstacles such as crossing the blood–brain barrier, which contribute to the difficulty in treatment. How to improve the remission rate, prolong survival, and improve the prognosis has always been an urgent problem for clinicians. Through a meta-analysis of existing research results, we found that ibrutinib can be considered a safe and effective treatment for CNSL, which may open a new avenues for CNSL treatment. However, due to the short treatment time, there are still several problems with ibrutinib as a treatment strategy for CNSL, such as the start and end time, frequency, duration, optimum combination regimen, and the eligible patient population for ibrutinib treatment. These issues need to be addressed in large-sample prospective randomized controlled trials. We expect that more comprehensive data will be available to help clinicians improve the application of ibrutinib as a treatment option for CNSL in the future.

## Data Availability Statement

The original contributions presented in the study are included in the article/**Supplementary Material**. Further inquiries can be directed to the corresponding author.

## Author Contributions

LL conceived the research design. LL, YW, and YC conducted literature search and data extraction. LL, XS, and QC performed the statistical analysis and contributed to writing of the report. XS and YL reviewed and edited the manuscript in detail. All authors contributed to the article and approved the submitted version.

## Funding

This work was supported by Capital’s Funds for Health Improvement and Research, NO.2020-2-2049.

## Conflict of Interest

The authors declare that the research was conducted in the absence of any commercial or financial relationships that could be construed as a potential conflict of interest.
