# Supplementary material for: Efficacy and Safety of Ibrutinib in Central Nervous System Lymphoma: A PRISMA-Compliant Single-Arm Meta-Analysis
Source: Front Oncol. 2021 Jul 1;11:707285. doi: 10.3389/fonc.2021.707285 (PMC8280788; doi:10.3389/fonc.2021.707285)
Supplement: Supplementary file 1 [file DataSheet_1.pdf]

Supplementary Table 1 The search strategy for each database.

Database	Search strategy
PubMed	1 Search: ((("central nervous system lymphoma"[Title/Abstract]) OR ("CNS lymphoma"[Title/Abstract])) OR ("CNSL"[Title/Abstract])) OR ("brain lymphoma"[Title/Abstract])
	2 Search: ("PCI 32765" [Mesh]) OR (((ibrutinib[Title/Abstract]) OR (imbruvica[Title/Abstract])) OR ("PCI 32765"[Title/Abstract]))
	3 Search: (((("central nervous system lymphoma"[Title/Abstract]) OR ("CNS lymphoma"[Title/Abstract])) OR ("CNSL"[Title/Abstract])) OR ("brain lymphoma"[Title/Abstract])) AND (("PCI 32765" [Mesh]) OR (((ibrutinib[Title/Abstract]) OR (imbruvica[Title/Abstract])) OR ("PCI 32765"[Title/Abstract])))
Embase	#1. 'central nervous system lymphoma':ab,ti OR 'cns lymphoma':ab,ti OR 'cnsl':ab,ti OR 'brain lymphoma':ab,ti<br>#2. 'central nervous system lymphoma'/exp<br>#3. 'brain lymphoma'/exp
	#4. 'ibrutinib'/exp<br>#5. ibrutinib:ab,ti OR imbruvica:ab,ti OR 'pci 32765':ab,ti
	#6. #1 OR #2 OR #3<br>#7. #4 OR #5<br>#8. #6 AND #7
Cochrane library	#1. ("central nervous system lymphoma"):ti,ab,kw OR ("CNS lymphoma"):ti,ab,kw OR ("CNSL"):ti,ab,kw OR ("brain lymphoma"):ti,ab,kw
	#2. (ibrutinib):ti,ab,kw OR (imbruvica):ti,ab,kw
	#3. #1 AND #2
Wanfang Data Knowledge Service Platform	1 (题名或关键词:(中枢神经系统淋巴瘤)+题名或关键词:(中枢系统淋巴瘤)+题名或关键词:(中枢神经淋巴瘤)+题名或关键词:(中枢性淋巴瘤))
	2 (题名或关键词:(伊布替尼)+题名或关键词:(依鲁替尼))
	3 (题名或关键词:(中枢神经系统淋巴瘤)+题名或关键词:(中枢系统淋巴瘤)+题名或关键词:(中枢神经淋巴瘤)+题名或关键词:(中枢性淋巴瘤))*(题名或关键词:(伊布替尼)+题名或关键词:(依鲁替尼))
China National Knowledge Infrastructure	(((关键词=中枢系统淋巴瘤 或者 keyword=中英文扩展(中枢系统淋巴瘤))或者(关键词=中枢神经淋巴瘤 或者 keyword=中英文扩展(中枢神经淋巴瘤)))或者(关键词=中枢神经系统淋巴瘤 或者 keyword=中英文扩展(中枢神经系统淋巴瘤)))并且((关键词=伊布替尼 或者 keyword=中英文扩展(伊布替尼))或者(关键词=依鲁替尼 或者 keyword=中英文扩展(依鲁替尼))) (模糊匹配)

2 Supplementary Figures

Supplementary Figure 2 Pooled partial response of central nervous system lymphoma.

Supplementary Figure 3 Pooled partial response of central nervous system lymphoma.

A Ibrutinib monotherapy. B Ibrutinib combined with chemotherapy.

Supplementary Figure 4 Pooled partial response of primary central nervous system lymphoma.

Supplementary Figure 5 Pooled partial response of primary central nervous system lymphoma.

A Ibrutinib monotherapy. B Ibrutinib combined with chemotherapy.

Supplementary Figure 6 Pooled partial response of SCNSL and r/r CNSL.

A secondary central nervous system lymphoma B refractory/relapsed central nervous system lymphoma

Supplementary Figure 7 Pooled partial response of r/r PCNSL and *MYD88+CD79B* WT.

A refractory/relapsed primary central nervous system lymphoma B both *MYD88* and *CD79B* wild-type

Supplementary Figure 8 Pooled partial response of *MYD88* mutation and *CD79B* mutation.

A pooled partial response of *MYD88* mutation. B pooled partial response of *CD79B* mutation.

Supplementary Figure 9 Survival of refractory/relapsed central nervous system lymphoma (r/r CNSL).

A 12-mouth overall survival. B 12-mouth progression-free survival. C

24-mouth overall survival. D 24-mouth progression-free survival.
