# Supplementary figures and images for: Efficacy and Safety of Ibrutinib in Central Nervous System Lymphoma: A PRISMA-Compliant Single-Arm Meta-Analysis
Source: Front Oncol. 2021 Jul 1;11:707285. doi: 10.3389/fonc.2021.707285 (PMC8280788; doi:10.3389/fonc.2021.707285)

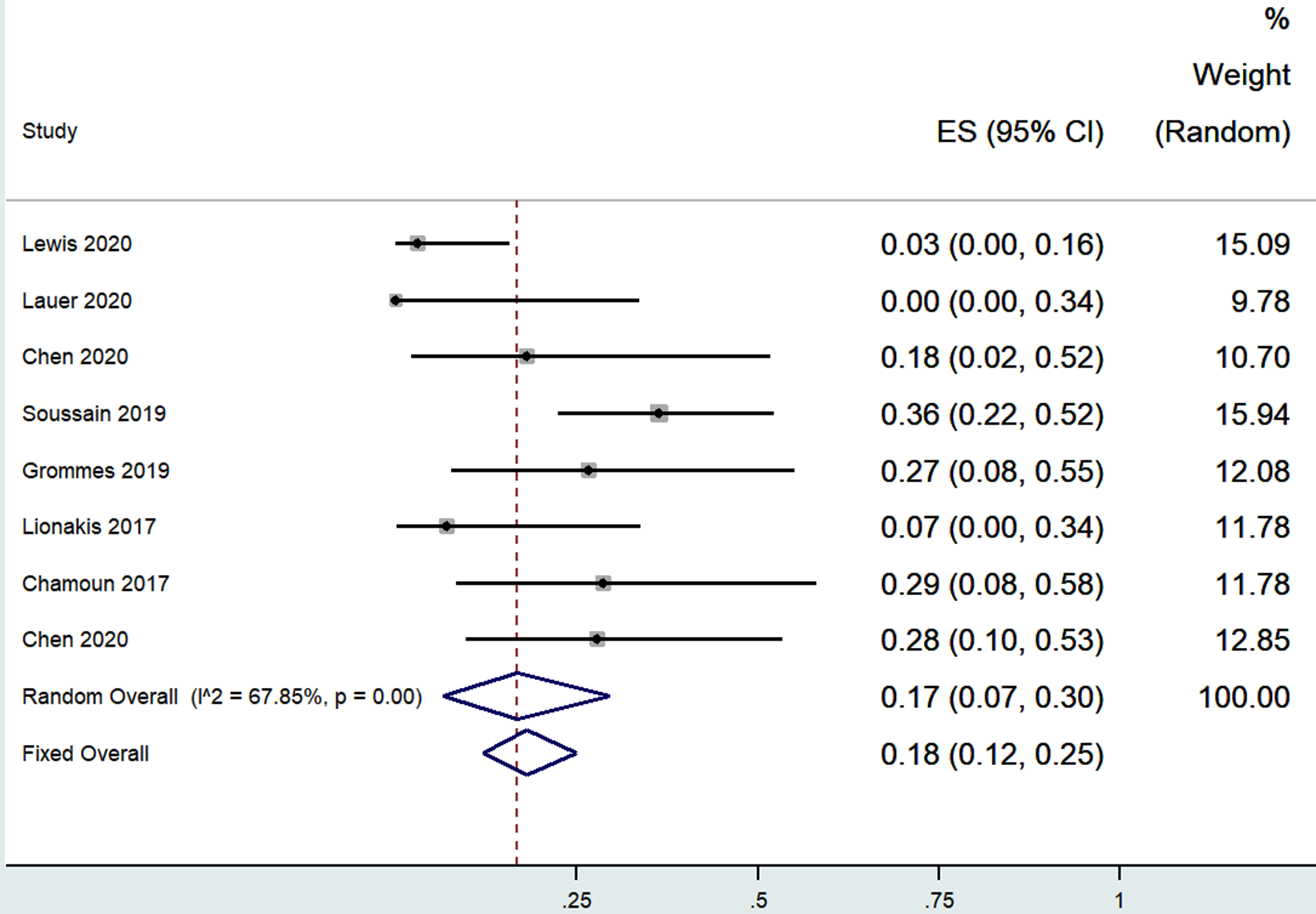

Supplement: Supplementary file 2 [file Image_1.tif]

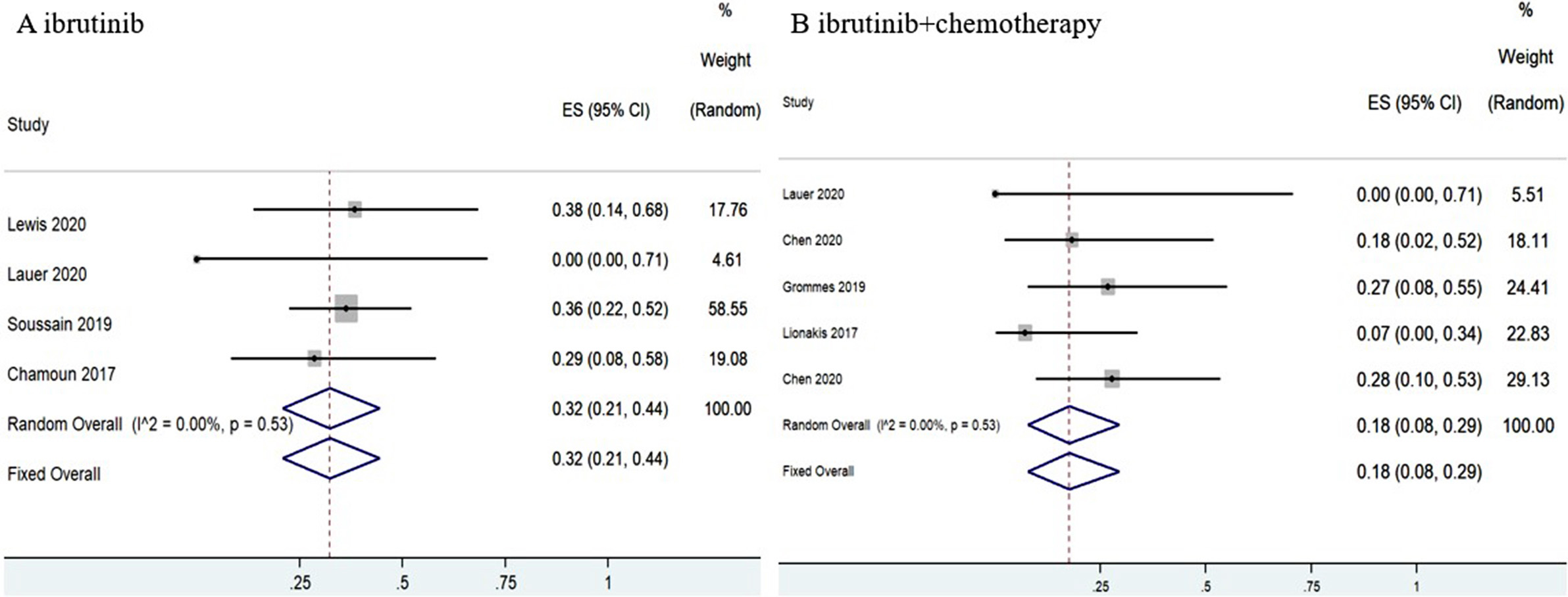

Supplement: Supplementary file 3 [file Image_2.tif]

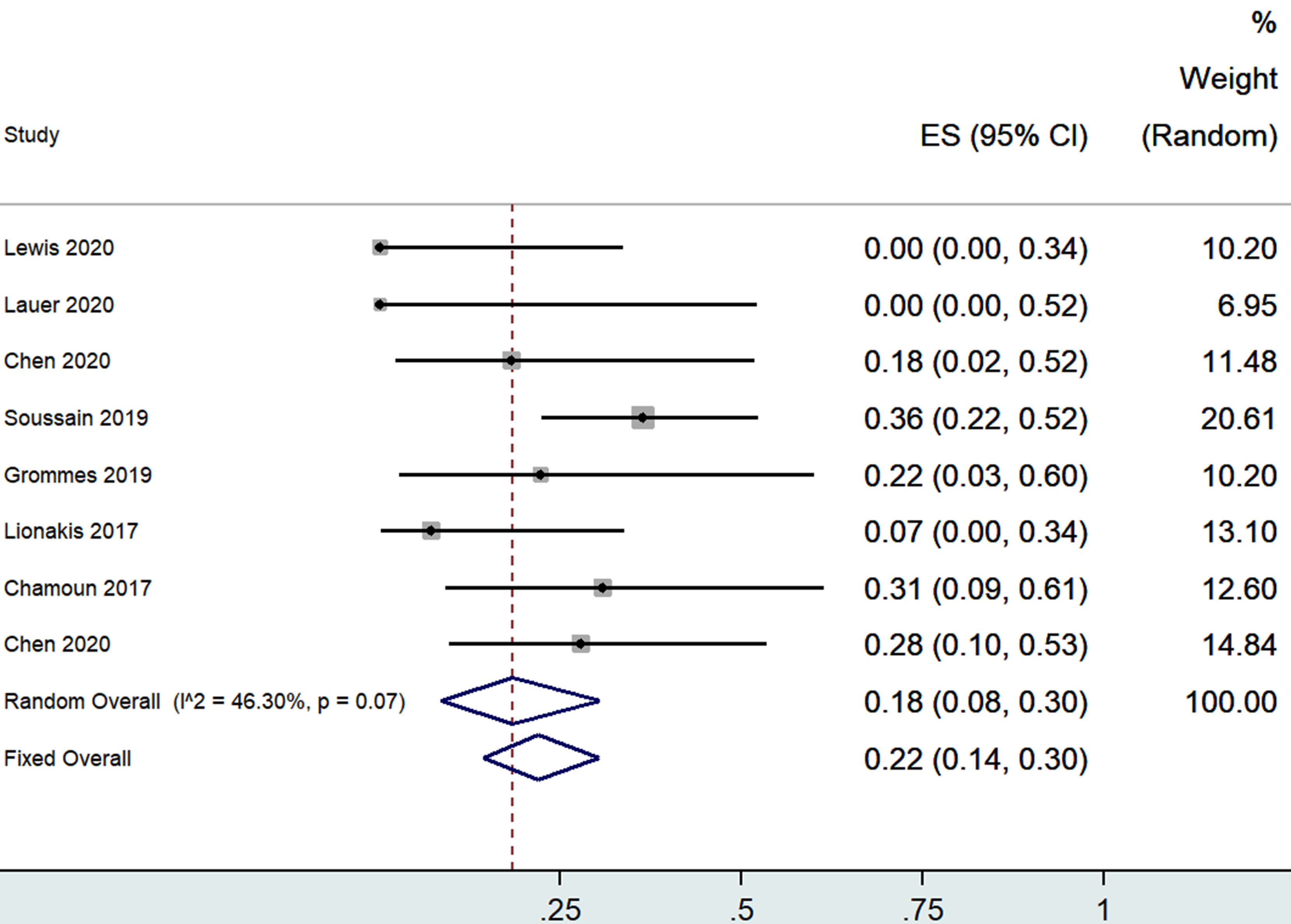

Supplement: Supplementary file 4 [file Image_3.tif]

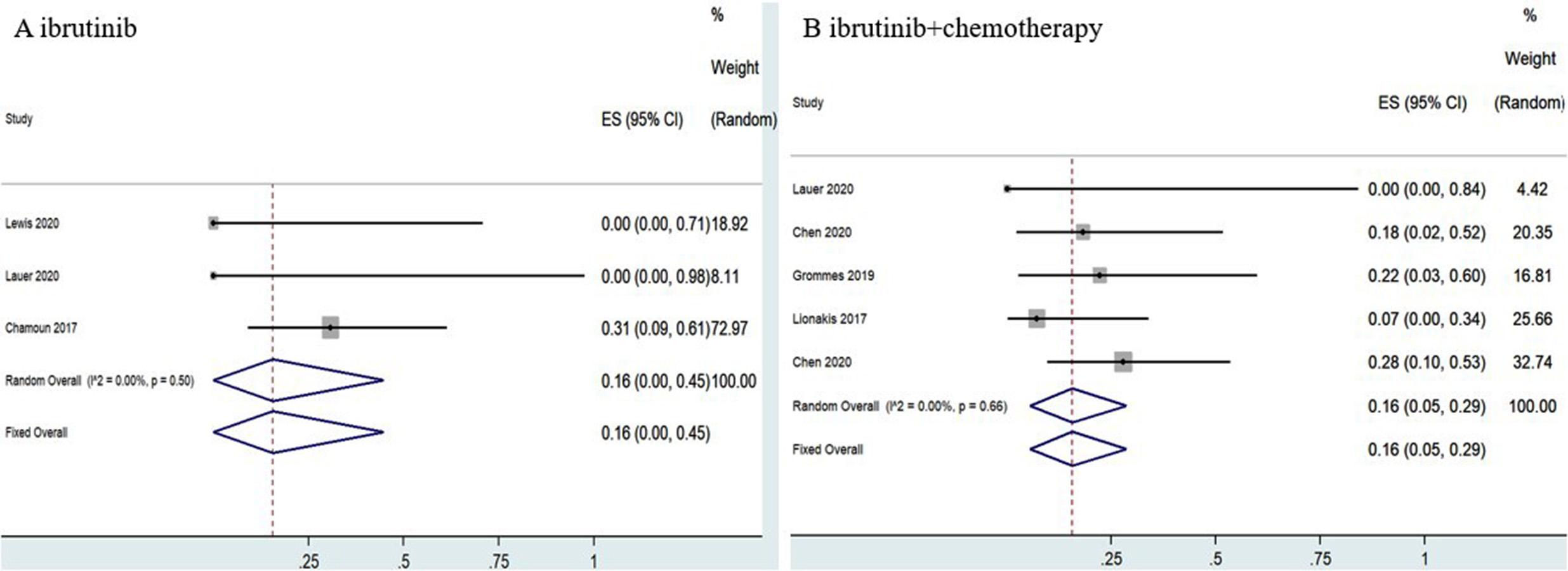

Supplement: Supplementary file 5 [file Image_4.tif]

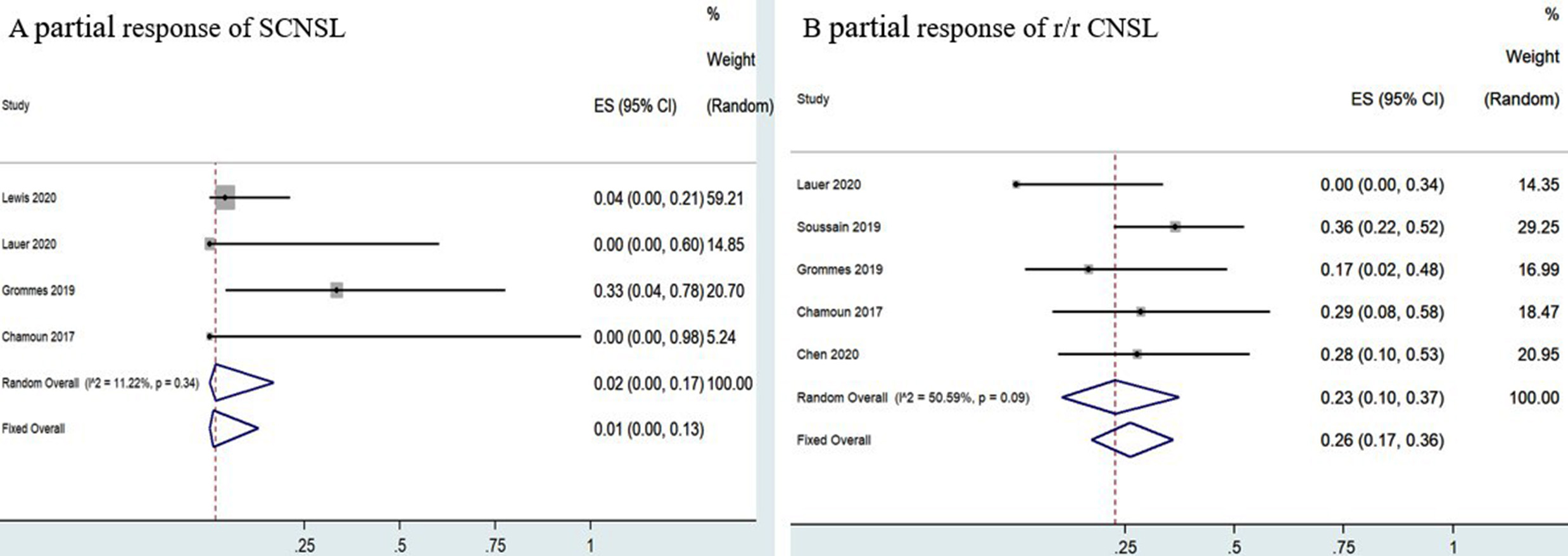

Supplement: Supplementary file 6 [file Image_5.tif]

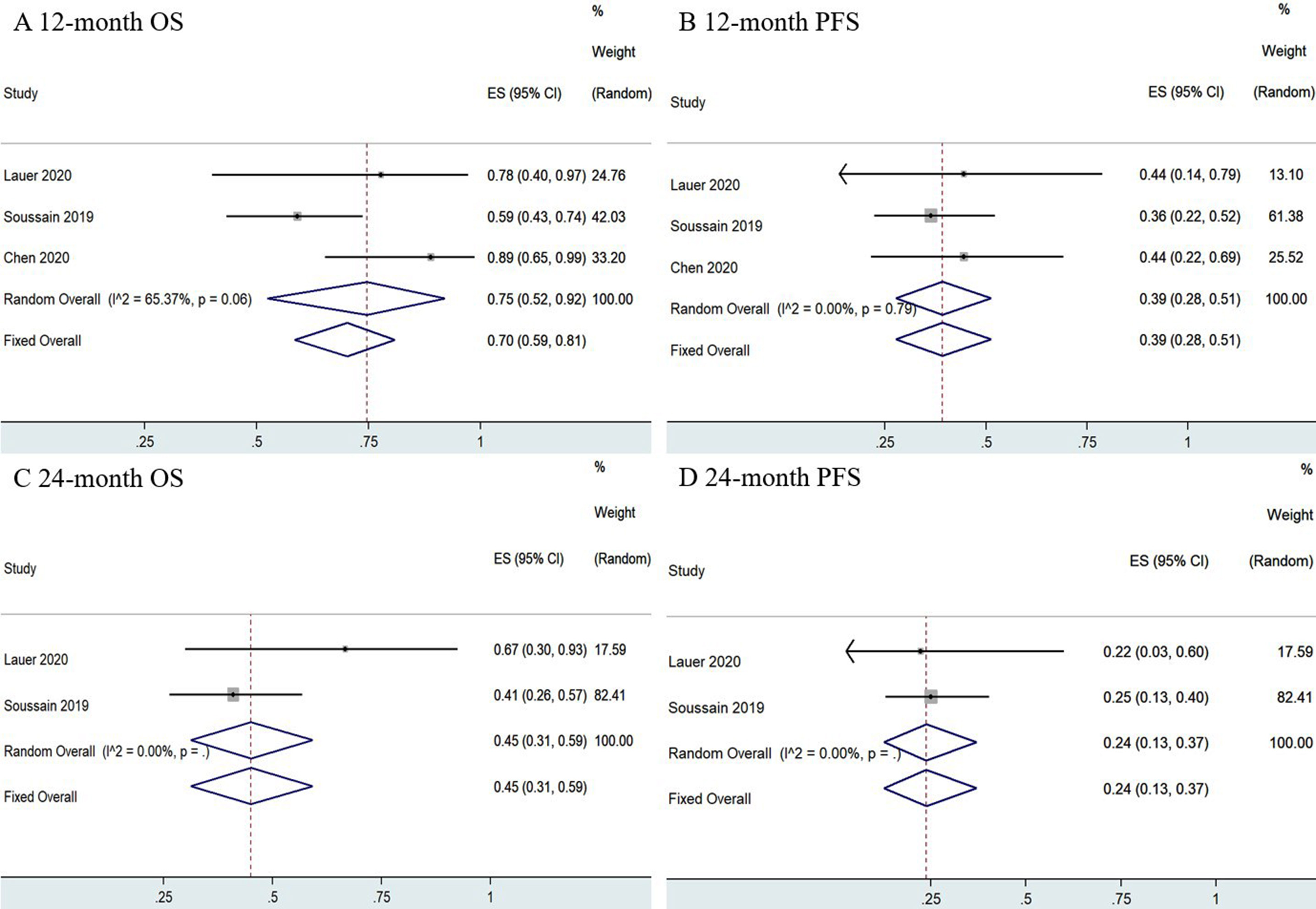

Supplement: Supplementary file 7 [file Image_6.tif]

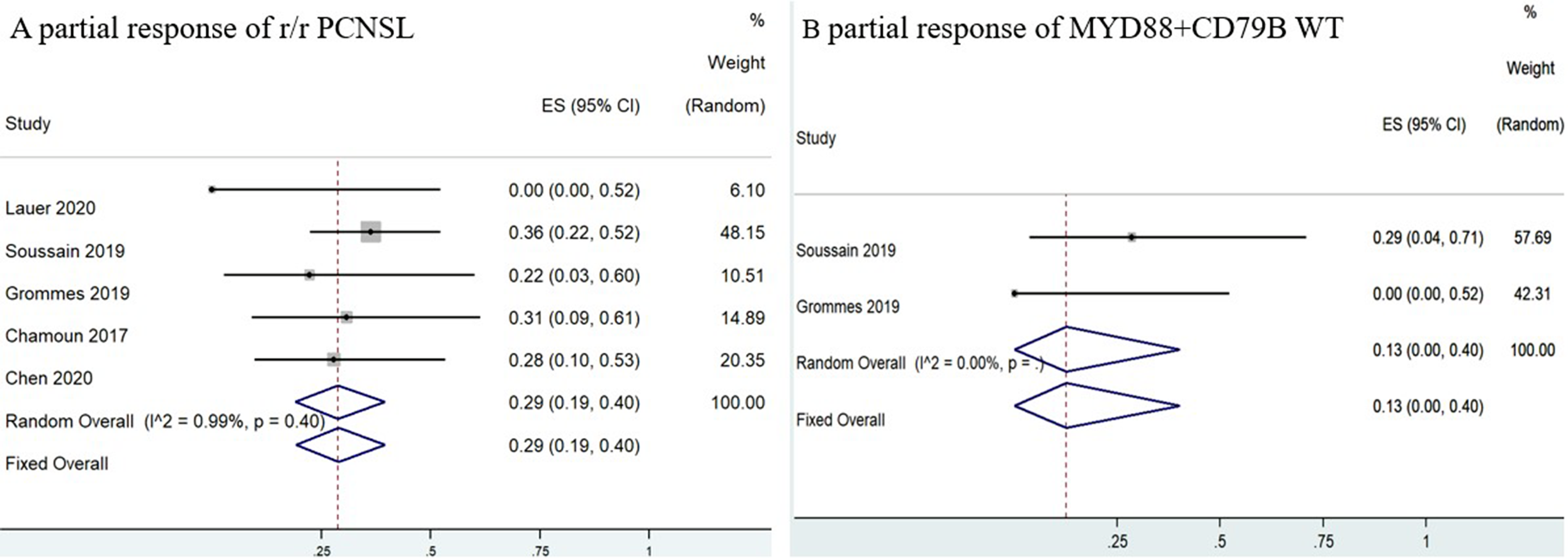

Supplement: Supplementary file 8 [file Image_7.tif]

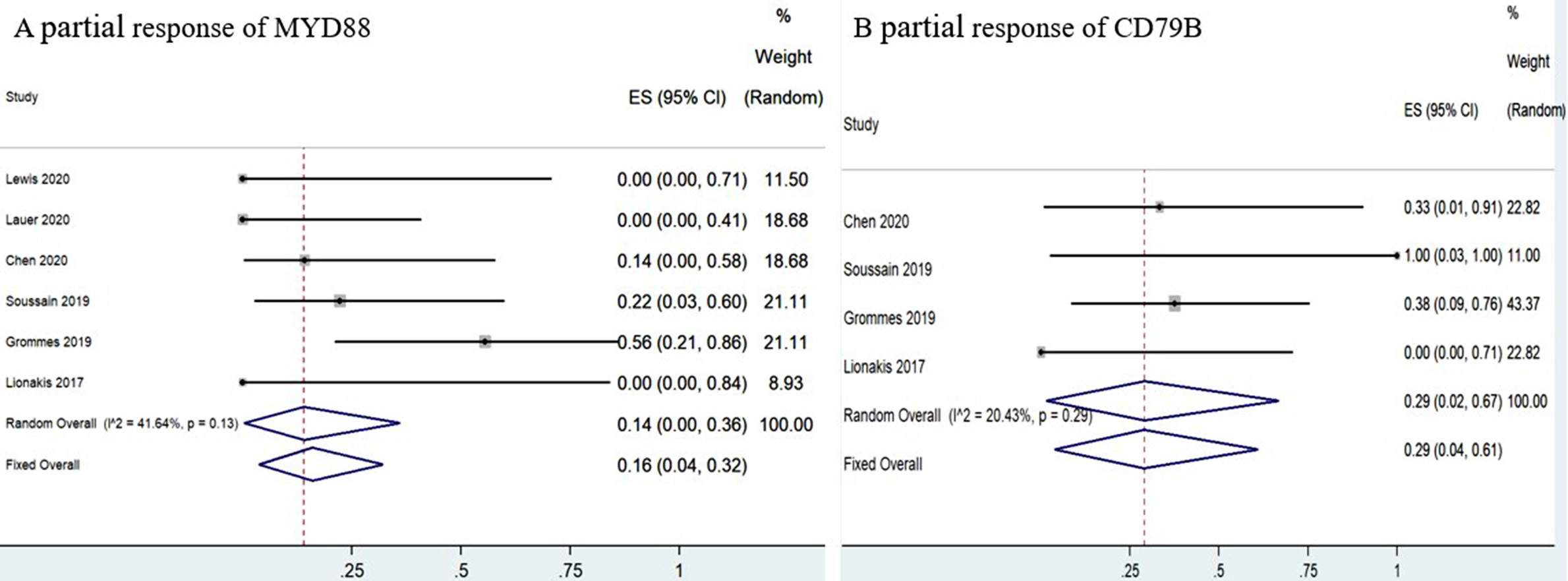

Supplement: Supplementary file 9 [file Image_8.tif]
